# Carroll County Medical Society

**Published:** 1869-07-15

**Authors:** J. Halder, Jno. L. Hostetter

**Affiliations:** Secretary; President


### *Carroll County Medical Society.*

The Society met pursuant to call of the Secretary at the Supervisor's rooms, in Mt. Carroll, at 11 o'clock, A. M., June 9, 1869. After making some arrangements for the good of the society, and exchanging views on medical topics, the Society adjourned until 2 o'clock, P. M.

Afternoon Session—Meeting called to order by the President; minutes of previous meeting read and approved. Dr. Thos. Winston, of Mt. Carroll, Illinois, and Dr. Nelson Rhinedollar, of Mt. Carroll, were then elected and admitted to full membership in the society.

On motion, it was ordered that the chair appoint a committee of three to draft a fee bill, and report at the next regular meeting. The following named members were appointed to read papers at our next meeting:

On Gun Shot Wounds—Dr. T. Winston, Foreston; on Contagious Diseases —Dr. D. M. Greely, Mt. Carroll; on Materia Medica—Dr. N. Rhinedollar, Mt. Carroll; on Medical Botany—Dr. Shimer, Mt. Carroll; on Diseases of the Liver—Dr. J Haller, Lanark; on Diseases of the Heart—Dr. J. B. Porter, Lanark. Some, who were present at this meeting, were not prepared. We shall be pleased to hear from them at our next.

On motion, it was ordered that any member or others, having papers prepared, and who cannot attend in person to read them, will please send them to the Secretary a few days before the time of meeting. Dr. Shimer then announced that Prof. Robley Dunlingson, M.D., of Jefferson Medical College, Philadelphia, died April 1, 1869. On motion of Dr. J. B. Porter, Dr.

Shimer was appointed to present a biographical sketch and eulogy on the name of our highly esteemed and deeply lamented brother and instructor, to be read at our next meeting. Dr. Greeley, of Mt. Carroll, read an essay on "Veratrum Viride," which, although brief, was well written. The Doctor set forth some very able arguments in regard to this agent. There was quite an interesting discussion of this subject by Drs. Skinner, J. B. Porter, Winston and Hostetter. Some of the members then gave reports of some cases in their practice, and asked for the views of others. This was very interesting, and if continued as a part of the exercises of our meetings, will, no doubt, be of very great benefit to the members and their patrons.

On motion of Dr. Greeley, it was decided to hold the next regular meeting of this society in Lanark, in the first part of September next; the day of meeting to be decided by the Secretary

On motion, it was ordered that the proceedings of this meeting be published in the county papers, and the MEDICAL JOURNAL of Chicago.

On motion, the society adjourned.

J. HALDER, M.D., *Secretary*,

JNO. L. HOSTETTER, M.D., *President*.

